# Exploring Mental Health and Development in Refugee Children Through Systematic Play Assessment

**DOI:** 10.1007/s10578-023-01584-z

**Published:** 2023-08-25

**Authors:** Katharina Bernhardt, Saskia Le Beherec, Jana Uppendahl, Marie-Anna Baur, Matthias Klosinski, Volker Mall, Andrea Hahnefeld

**Affiliations:** 1 Technical University of Munich, Munich, Germany; 2 kbo Kinderzentrum, Munich, Germany

**Keywords:** Child, Displacement, Migration, Trauma, Development, Play observation

## Abstract

To evaluate a standardized play observation as a measure of young children’s mental health and development in a clinical and refugee population. We conducted individual play observations with 70 refugee children aged 3- to 6-years and compared them to a clinical group of 111 age-matched children regarding their level of play development, social interaction during play, traumatic re-enactments, and emotionless-cold play. Additionally, we assessed children’s mental health, social-emotional development and markers of adversity by parent and educator report as well as their IQ-test scores and learning performance and related these factors to the play variables. Play variables were significantly correlated with IQ-test scores (r = 0.184, p = 0.037), learning performance (r = 0.208, p = 0.010) and vocabulary (r = 0.208, p = 0.021) in the comparison group and with social-emotional development in educator report (r = 0.368, p = 0.011), time spent in Germany (r = 0.342, p < 0.001) and parental distress (r = − 0.292, p = 0.034) in the refugee group. Children with more parent-reported adverse experiences showed less social-interactive play in the overall sample (r = − 0.178, p = 0.011). Our child-centered approach to standardized play observation augments information obtained from parent and educator reports and can provide valuable insights in subgroups where other commonly used tests are not available or applicable.

## Introduction

Forced displacement has become an increasingly prevalent issue in recent years, affecting over 36 million children in 2021 alone [47]. Given the elevated risk of accumulating adversities in the course of displacement, collecting data on young children’s mental health and development is highly relevant, though it is challenging due to their limited ability to verbally articulate experiences and symptoms [16, 43]. This has lead researchers and clinicians to rely on parental reports [24, 28, 29], although these may be influenced by the parent’s own symptomatology [2, 16, 28, 29]. Supplementary reports by educators are recommended to enhance sensitivity [28, 29], but limited access to daycare makes it difficult to obtain a multi-context perspective in refugee settings [5]. Play observations offer a viable solution to these challenges [10, 12, 13, 22]. When fundamental needs such as security, nourishment, and sleep are fulfilled [19, 32], play provides a natural setting for children to express and regulate their emotions, build social skills and acquire language and cognitive abilities [13, 30, 32]. Despite cultural variations in the interpretation and context of free play [9], play is governed by a homeostatic principle and is assumed to reflect the child’s overall progression and mental health [22, 32] based on an established chronological sequence of play development [8].

Forced displacement poses significant challenges for young children’s mental health and development [6, 45], including their abilities and opportunities to engage in complex play [1, 6, 19, 39, 45]. Previous studies have described less developed and social-interactive play behaviors in war-exposed and refugee children due to deprivation and elevated stress [12]. Moreover, re-enactments of traumatic events and restriction in affect during play were shown to be indicative of PTSD and psychological distress [12, 13, 42] that was not detected in parent reports [2, 3, 7]. Despite the significance of play observations in assessing young children’s development in challenging circumstances [37, 50], no standardized play measures have been utilized to examine developmental trajectories in refugee populations. The aim of this empirical study was to evaluate the effectiveness of a structured play observation in examining mental health, social-emotional and cognitive development of young children with and without refugee experiences and relate play outcomes to markers of adversity (flight duration, time in Germany, time in childcare, number of adverse experiences, parental mental health).

The following hypotheses were tested:

- **H1.1** We expect refugee children to show lower levels of play development and social interaction during play, and a higher rate of reenacting and emotionless play behavior compared to a clinical comparison group without refugee experience.
- **H1.2** We expect markers of adversity to correlate negatively with play development and social interaction during play, and positively with reenacting and emotionless play.
- **H2.1** Reenacting and emotionless play, along with lower levels of play development and social interaction during play is associated with children’s mental health.
- **H2.2** Levels of play development and social interaction during play are associated with social-emotional development, cognitive and learning performance.

## Methods

### Study Design and Population

This article was part of the InterCuLtUral Child DEvelopment Project (INCLUDE), design and methods were adapted from previously published studies [28, 29]. The cross-sectional study was conducted between June 2021 and August 2022 in two reception camps for refugees in Munich, Germany. Data was collected directly in the camps in separate examination rooms. Children aged 3- to 6-years who were born outside of Germany and were attended by at least one parent were included in the study. Eligible families were contacted in the camps, received information about the study and were invited to an appointment, with interpreters if necessary.

An age-matched comparison group of children who were referred due to developmental, language and/or social-emotional problems was recruited and examined at the Social Pediatric Center (SPZ Schwabing) in Munich. All children who were present in the center from January to April 2022 were informed about the study and asked to participate. Children with diagnoses of serious somatic or developmental disorders, intellectual impairment and autism were excluded.

### Study Procedure

The study protocol was approved by the ethics committee of the Medical Faculty of the Technical University Munich. Informed written consent was given by all parents prior to data collection. They were asked for general information on age, education, medical history, language, and cultural background as well as duration of flight and time since arrival in Germany. Children’s play behavior was observed by the investigators while parents filled out the questionnaires in their native language (PORTA) [46] or with the help of interpreters. Families were remunerated for their participation with coloring books and pens for the children and received medical reports on the psychometric study results. Educators were asked to fill out questionnaires about the participating children.

### Assessment

#### Play Observations

All children were offered the same set of toys in a defined arrangement including dolls, cookware, a country farm set and building blocks. The investigators did not give any instructions, allowing children to play naturally. Play sequences of ten minutes were videotaped, observed by two trained investigators and rated according to a self-developed observational measure adapted from several established scales [30, 33]. Children’s play development was coded as follows: sensorimotor, functional/exploratory, construction play, symbolic play, role-play [30, 32]. Based on previous literature about play behaviors of war-exposed and traumatized children [11, 12, 33] and clinical experience, we created an index of social interaction that indicates children’s initiative, perseverance, interaction and language use during play. Emotionless play behavior and re-enactments of traumatic events as indicators of posttraumatic stress were coded separately. Regular play-coding sessions were held with the investigators to ensure a standardized procedure regarding the coding of play observations. Analyses of interrater reliability yielded satisfactory results for all sections, apart from the reenactment item (see Table 1).

**Table 1:** Inter-rater reliability of the self-developed play observation sheet

	κ	ICC
Play development	–	0.809
Social interaction	–	0.825
Emotionless play	0.796	–
Reenactments	–	–

Reliability analysis for the reenactment item could not be performed due to missing variance within the item

#### Children’s Mental Health

Parents filled out the Child and Adolescent Trauma Screening (CATS; [41]), a validated and open-access screening tool designed for assessing potentially traumatic experiences and stress reactions according to DSM-5 [4] criteria for PTSD. We used the recommended cut-off value of ≥ 16 as an indication for a clinically relevant level of stress symptoms.

The Strengths and Difficulties Questionnaire (SDQ; [26]) was administered to all parents and educators in the corresponding form. It is a fully evaluated, behavior-oriented screening questionnaire for 2- to 17-year-old children and adolescents comprised of 25 items on children’s emotional problems, behavioral problems, hyperactivity, peer problems and prosocial behavior. A total difficulties score can be calculated, with results of ≥ 16 being considered as clinically relevant.

#### Children’s Social-Emotional Development

The subscale for social-emotional competences from the Behavior Observation Scale for Preschool Children, standardized and evaluated for 3- to 6-year-old children (Verhaltensbeurteilungsbogen für Vorschulkinder; VBV-ER 3-6; [17]), was filled out by the educators. Children’s competences regarding interaction and communication, conflict resolution, and play intensity were rated on a five-point Likert-scale.

#### Children’s Cognitive Development

The nonverbal version of the Kaufmann Assessment Battery for Children (KABC-II: SFI; [36]) was conducted with refugee children to assess their cognitive testing abilities. The KABC-II has good psychometric properties concerning construct validity and reliability and has been developed as a language-free and culture-fair diagnostic instrument [36]. The comparison children were either assessed with the KABC-II or with a similar standardized language-free IQ test (WPPSI-IV, WNV, SON 2-7) and completed the subtest Vocabulary of the KABC-II, where children were asked to name pictures in German. Additionally, the subtest Atlantis of the KABC-II was conducted with all children to assess their short-term learning performance.

#### Parent Mental Health

Parental mental health was assessed in both groups with the Refugee Health Screener (RHS-15; [51]), a culturally sensitive and empirically validated screening tool. The RHS captures symptoms associated with various psychiatric diseases and was developed with and for individuals with refugee experiences. A cut-off score of ≥ 12 or a distress thermometer score of ≥ 5 indicates a positive screening.

### Statistical Analysis

All statistical analyses were performed with SPSS 28.0 for Windows (IBM [31]).

Due to violations of normality and homogeneity of variances assumptions in our data, we conducted non-parametric Mann–Whitney-*U*-Tests to compare play development and social interaction during play in groups of refugee and non-refugee children, and used correlations (Spearman rank correlation, one-tailed) to investigate associations between play variables, trauma-related stress symptoms, social-emotional outcomes as well as cognitive and learning performance and markers of adversity. To investigate possible age-related effects on children’s play development, we conducted Kruskal–Wallis and subsequent Mann–Whitney-*U*-tests with age as group factor.

## Results

### Participants

Characteristics of the study population are described in Table 2.

**Table 2:** Demographic and symptom characteristics of the samples

	Refugee group	N_refugee_	Comparison group	N_comparison_
Mean age in years [CI]	4.94 [4.66–5.22]	70	5.20 [5.00–5.40]	111
Percent female (%)	34 (49)	70	32 (29)	111
Parental education in years [CI]	6.20 [4.62–7.78]	53	11.55 [10.82–12.28]	91
Number of literate mothers (%)	36 (51)	67	104 (94)	110
Day-care in hours/week [CI]	7.96 [5.90–10.02]	64	30.95 [28.82–33.09]	110
CATS				
At least one event reported (%)	62 (89)	67	28 (25)	110
Number of events reported [CI]	4.37 [3.77–4.98]	68	0.47 [0.28–0.66]	110
Mean number of symptoms [CI]	15.83 [12.81–18.85]	60	7.79 [3.68–11.90]	24
Children above threshold (%)	28 (40)	60	3 (3)	24
Full DSM-V criteria for PTSD (%)	23 (33)	61	4 (4)	24
SDQ parent				
Total difficulties [CI] *Above threshold (%)*	22.52 [20.93–24.12] *55 (79)*	65	19.79 [18.77–20.81] *76 (69)*	111
SDQ educator				
Total difficulties [CI] *Above threshold (%)*	17.00 [15.56–18.41] *24 (34)*	45	19.42 [17.94–20.90] *45 (41)*	64
VBV				
Social-emotional competences [CI] *Below normal range (%)*	40.08 [34.73–45.43] *11 (16)*	39	41.78 [36.83–46.73] *14 (13)*	39
KABC-II				
IQ-testing performance [CI]	81.1 [77.06–85.14]	61	90.03 [86.65–93.41]	101
Learning performance [CI]	6.42 [5.79–7.10]	66	8.98 [8.37–9.59]	102
Vocabulary [CI]	–	–	6.18 [5.54–6.83]	100
RHS				
Parents above threshold (%)	56 (80)	66	20 (18)	111
Play behavior				
Play development [CI]	5.59 [5.24–5.94]	70	5.64 [5.33–5.95]	104
Social interaction [CI]	4.24 [3.89–4.59]	69	4.77, [4.46–5.08]	102
Emotionless-cold play (%)	1 (1)	68	2 (2)	103
Reenactments (%)	0 (0)	68	1 (1)	103

*CATS* Childhood and Adolescent Trauma Screening, *SDQ* Strength and Difficulties Questionnaire, *VBV* Behavior Observation Scale for Preschool Children, *KABC-II* Kaufmann Assessment Battery for Children, *RHS* Refugee Health Screener

In the refugee group, 70 children were analyzed. Children’s mean age was 4.94 years, mean duration of time since arrival in Germany was 3.71 months (*CI* 3.03–4.39), after an average flight duration of 29.69 months (*CI* 24.51–34.87). Families were from Afghanistan (84%), Kongo (4%), Venezuela (4%), Bolivia (1%), Yemen (1%), Palestine (1%), Sierra Leone (1%), and Uganda (1%). Twenty (29%) children were born during flight. Most families were still in the process of asylum application (86%), 11% had a permanent residence permit and one family had a temporary halt to the deportation process. The 111 children of the comparison group were referred to the SPZ by educators, teachers, or parents. After medical and psychological examination, 32.4% received a diagnosis of combined developmental disorders (F83), 35% of speech or motor developmental disorders (F80, F82), and one child received a high-functioning Asperger diagnosis. Further, 43% received a psychiatric diagnosis (F43, F51, F90-F98) and 6% of comparison children did not receive a diagnosis. 78% of comparison children had a migration background with at least one parent born outside of Germany.

Data on children’s and parents’ mental health were obtained for 168 (92%) participants. Play assessments could not be obtained for 8 (4%) children, since they did not interact with the provided play material. Data on cognitive development were incomplete for 28 (15%) children, because IQ tests were not conducted within the study’s timeframe for the clinical comparison group. We received complete educator ratings for 39 children in the refugee group (56%) and for 40 children in the comparison group (36%). The children without complete educator rating had lower rates of maternal literacy (*U* = 3265.5, *Z* = − 2.358, p = 0.018, r = 0.18) but did not differ in any other outcome variables.

### Test Results

In the clinical comparison group, there was a significantly higher rate of boys (*U* = 3118.00, *Z* = − 2.680, *p* = 0.007, r = 0.20) and alphabetized mothers (*U* = 2181.00, *Z* = − 6.495, p < 0.001, r = 0.49), more years of parental education (*U* = 1134.50, *Z* = − 5.300, p < 0.001, r = 0.44), and more hours per week in day-care (*U* = 615.00, *Z* = − 9.102, p < 0.001, r = 0.69).

Parents of refugee children reported a higher rate of potentially traumatic experiences (*U* = 643.00, *Z* = − 9.907, p < 0.001, r = 0.74) and trauma-specific symptom load for their children (*U* = 427.50, *Z* = − 2.902, p = 0.004, r = 0.32) as well as a higher symptom load for themselves (*U* = 655.50, *Z* = − 9.133, p < 0.001, r = 0.69) than parents in the comparison group. Refugee children had significantly higher Total Difficulties scores (*U* = 2718.50, *Z* = − 2.729, p = 0.006, r = 0.21) on the SDQ by parent report but scored significantly lower than comparison children for Total Difficulties by educator report (*U* = 1117.50, *Z* = − 1.990, p = 0.047, r = 0.19).

There were no group differences on the VBV (*U* = 714.50, *Z* = − 0.460, p = 0.646, r = 0.05). Refugee children scored lower than the clinical comparison group on measures of short-term learning (*U* = 1838.00, *Z* = − 4.986, p < 0.001, r = 0.38) and IQ test performance (*U* = 2100.50, *Z* = − 3.389, p < 0.001, r = 0.27).

#### H1.1 Group Differences

Refugee children scored similarly to comparison children regarding their play development (*U* = 3516.00, *Z* = − 0.38, p = 0.701, r = 0.03), but significantly lower on social play (*U* = 2841.00, *Z* = − 2.142, p = 0.032, r = 0.16). For play development, there was a significant effect for age in the comparison group (*Kruskal–Wallis-H* = 13.612, p = 0.003), but not in the refugee group, with post-hoc analyses indicating that 3-year-old children played at a significantly less advanced level than 4- to 6-year-old’s (*U*_3–4_ = 93.00, *Z* = − 3.51, p < 0.001, r = 0.52; *U*_3–5_ = 163.00, *Z* = − 2.77, p = 0.006, r = 0.38; *U*_3–6_ = 110.50, *Z* = − 2.85, p = 0.004, r = 0.43).

Gender differences emerged in play development among refugee children, with girls displaying higher levels of play development than boys (*U* = 441.00, *Z* = − 2.03, p = 0.042, r = 0.24).

#### H1.2 Play as Indicator of Children’s Exposure to Adversity

In the whole sample, children with more adverse experiences in parent rating showed less social-interactive play (r = − 0.178, p = 0.011). This effect did not reach significance when both groups were analyzed separately.

Refugee children’s play development was positively correlated to time since arrival in Germany, and refugee children’s social interaction during play was associated with parent’s symptoms (Table 3).

**Table 3:** Pearson correlations of children’s play behavior with markers of adversity

	Group	Flight duration	Time in Germany	Time in daycare	CATS events	RHS
Play development	Comparison	–	–	0.073	0.065	0.120
	Refugee	− 0.041	0.342**	0.019	0.135	0.022
Social interaction	Comparison	–	–	− 0.021	− 0.067	0.034
	Refugee	0.088	0.143	0.113	− 0.077	0.266*

*CATS* Child and Adolescent Trauma Screening, *RHS* Refugee Health Screener

*p < 0.005, one-tailed

**p < 0.001, one-tailed

Parent ratings of their own and the children’s trauma-specific and general symptom load were significantly correlated in the refugee group (r_RHS-CATS_ = 0.506, p < 0.001; r_RHS-SDQ_ = 0.477, p < 0.001), and the comparison group (r_RHS-CATS_ = 0.354, p = 0.049; r_RHS-SDQ_ = 0.415, p < 0.001).

#### H2.1: Play Behavior and Children’s Mental Health

In the comparison group, one child showed reenacting themes, and two children displayed emotionless cold play behavior, as did one child in the refugee group. There were no significant correlations between play variables and trauma-related stress symptoms or general symptom load in parent and educator rating for both groups (see Table 4).

**Table 4:** Spearman correlations of children’s play and social interaction scores with trauma-related and general symptom load

	Group	Social interaction	CATS symptoms	SDQ-Parent	SDQ-Educator
Play development	Comparison	0.379**	0.088	− 0.040	0.060
	Refugee	0.320**	− 0.015	0.062	0.163
Social interaction	Comparison		0.344	0.077	0.151
	Refugee		− 0.094	0.010	0.151

*CATS* Child and Adolescent Trauma Screening, *SDQ* Strength and Difficulties Questionnaire

*p < 0.005

**p < 0.001

Parent and educator ratings of the children’s general symptom load were not correlated (r = 0.111, p = 0.128), but we found high intercorrelations for educator ratings concerning children’s scores on the VBV and the SDQ (r = − 0.295, p = 0.005).

#### H2.2: Play Behavior and Development

**Fig. 1:**
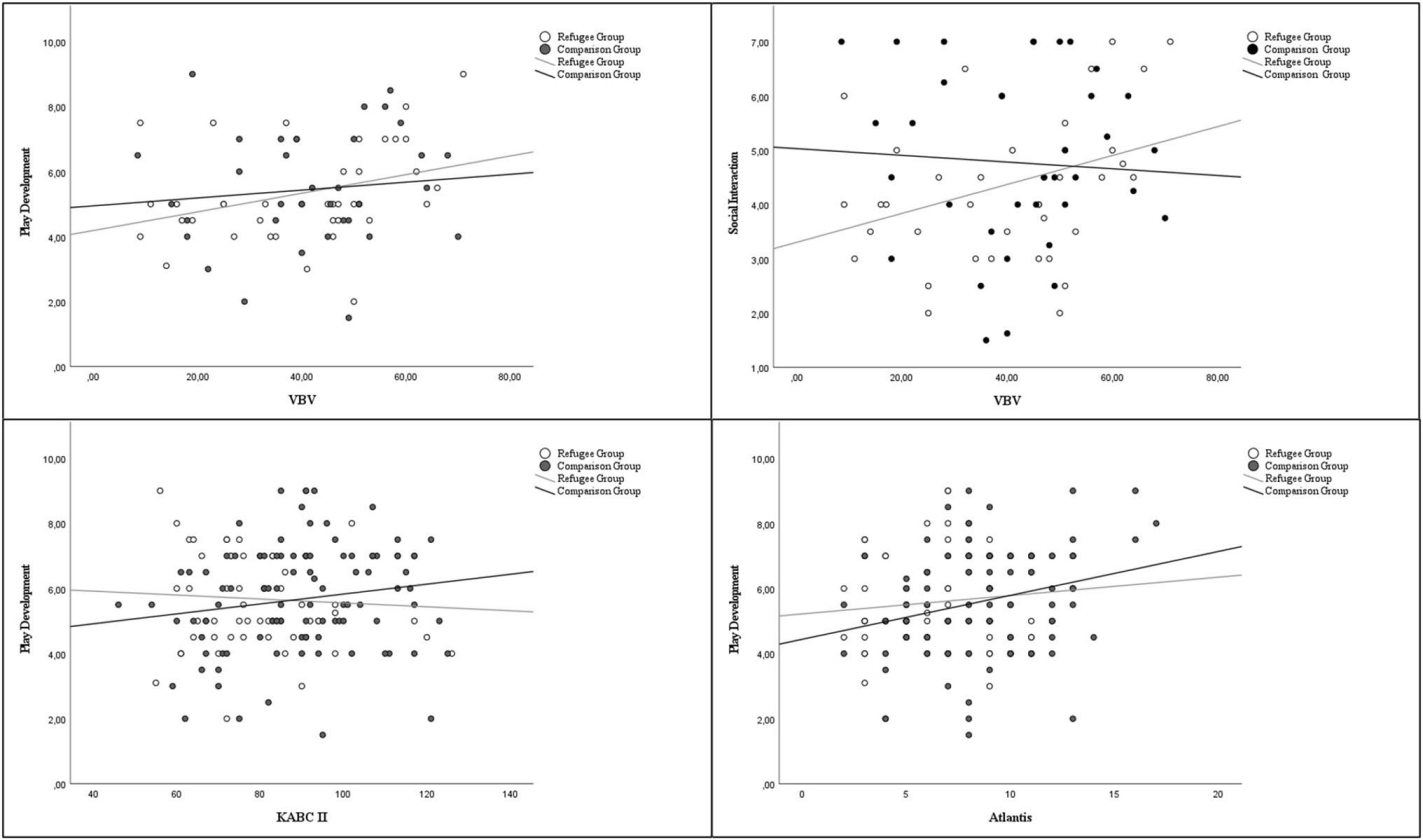
Scatter Plots for play variables and social-emotional competencies in educator rating (VBV), IQ-testing performance (KABC-II) and learning performance (Atlantis)

In the comparison group, play development correlated with IQ test performance, learning performance and vocabulary, while no corresponding correlations emerged in the refugee group. A significant correlation was found between play measures and VBV scores in the refugee group, but not in the comparison group (see Table 5, Fig. 1).

**Table 5:** Spearman correlations of children’s play and social interaction scores with developmental outcomes

	Group	Non-verbal IQ	Learning performance	Vocabulary	VBV
Play development	Comparison	0.184*	0.232*	0.208*	0.167
	Refugee	− 0.061	0.073	–	0.368*
Social interaction	Comparison	0.012	0.054	0.215*	0.018
	Refugee	0.002	0.025	–	0.318*

*VBV* Behavior Observation Scale for Preschool Children

*p < 0.005

**p < 0.001

## Discussion

Our study findings highlight both similarities and differences in play behaviors of young children with and without refugee experiences.

While play development was comparable in both groups, advanced play was observed more frequently in older children from the comparison group, whereas no age-related effects emerged among refugee children. We did not observe a higher frequency of reenacting and emotionless cold play behavior in refugee children, but lower social interaction scores compared to the comparison group. Therefore, *Hypothesis 1.1* was partly verified.

As hypothesized, children with more parent-reported adverse experiences showed less social play in the overall sample, underscoring the potential impact of adversity on children’s play. This effect is particularly relevant for refugee populations, who are at heightened risk of exposure to cumulative adversities, as shown in our study. Parental distress was identified as indicator for children’s general and trauma-related symptom load in both groups, and significantly correlated with refugee children’s social play. This is in line with previous findings that potentially traumatized parents may struggle to provide their children with a sense of security and stability [48, 49], affecting both children’s mental health and engagement in play [14, 25, 28, 29, 40]. Childcare is known to serve as protective measure for children facing adversity [27, 38], which could explain why the correlation between parental distress and children’s social play was significant only in the refugee group without regular childcare enrollment. This may also partially explain the lower level of social play, as well as the absence of age-related effects on play development in the refugee group. While time spent in childcare was not correlated with either play measure, this is possibly due to the little variance in time children spent in childcare, which consequently rendered additional covariate analyses in our study unfeasible. Moreover, childcare in the refugee sample consisted of playgroups within the camps that may differ from the regular and stable institutions the comparison children were enrolled in. Nonetheless, the positive correlation between the duration of stay in Germany and social play implies that a more secure environment can promote play and social behavior. Taken together, these findings indicate that it is not solely the experience of displacement itself, but rather the exposure to adverse experiences and environmental factors such as parental functioning and access to childcare that significantly impact a child’s engagement in play and overall healthy development, as supported by previous empirical literature [6, 12].

Our data revealed no associations between parent ratings of children’s trauma-related and general symptom load and markers of child mental health from other perspectives such as systematic play observations or educator assessments, leading us to reject *Hypothesis 2.1*. Consistent with prior research [28, 29], it moreover suggests that parents might have overestimated their children’s distress, underscoring the need for complementary perspectives of children’s symptomatology.

Our standardized play observation effectively mapped short-term learning, IQ test performance, and language abilities in a help-seeking population born and raised in Germany. However, no corresponding relationships were found in the group of refugee children. This aligns with previous findings indicating that standardized tests designed for Western populations may not be valid measures for cognitive development in children from diverse ethnic and social backgrounds [23, 28, 29, 35]. The social interaction score was significantly correlated with language development in the comparison group. Given that refugee children demonstrated less social play, this may imply that they also exhibit lower language proficiency, which was already below average in our comparison group. However, assessing refugee children’s language development was not feasible within the scope of this study and requires future research.

Our study found strong correlations between play observations and social-emotional competences in educator rating, suggesting effective measurement of social-emotional development in young refugee children. Notably, no corresponding correlations emerged with general symptom scores, suggesting that strength-based measures and our play observations may more accurately assess children’s psychosocial competences than deficit-focused measures [15, 44]. Solely assessing children’s symptom load may underestimate children’s abilities, as social-emotional competences in a specific context may not equate to the absence of problem-based behavior [44]. Our findings highlight both difficulties and social-emotional abilities of refugee children, setting a focus on their resilience in the face of refugee accommodations in which the educator reports where obtained [34].

### Strengths and Limitations

Despite marked effort, we only obtained 56% educator reports for refugee children and 36% for comparison children, which may have affected the accuracy of our findings. However, it is essential to note that the study took place during the pandemic lockdown, which limited daycare availability. It also highlights the challenge faced by refugee children in accessing stable educational and play environments [1, 19], emphasizing the need for child-centered assessment methods.

In contrast to previous studies reporting high rates of reenacting play in traumatized and war-exposed preschool-age children [2, 12], we did not observe any such play themes in our sample. This discrepancy may result from differences in play measures used, as previous studies aimed to capture posttraumatic play, while we observed children’s spontaneous and undirected play with neutral materials. While our defined duration of ten minutes for play sequences may not have allowed enough time for the children to engage in reenacting play, the observed absence of such play themes with neutral play materials may suggest the children’s resilience in a stable environment. Previous literature has shown that different types of traumatic events may impact children’s play in distinct ways. In a study with war-exposed children, specific experiences such as the loss or injury of parents negatively influenced various aspects of children’s play activity, but not the severity of the terror events [12]. Future studies with larger sample sizes should explore the impact of different types of traumatic events on children’s play in more depth.

The differing gender distributions within the groups could have influenced our results. While the literature suggests no gender differences in play development [21, 32], and our play materials appealed to both genders, our study found that girls had higher levels of play development than boys in the refugee group. This might be related to cultural and social factors that shape gender roles and expectations in different communities.

In the refugee group, we had a 49% rate of illiterate mothers and parents had a significantly lower educational level than parents in the comparison group. Parental educational level has been consistently identified as important predictor of children’s developmental and behavioral outcomes [18, 28, 29]. Considering the lack of formal education among the children in the refugee group, it is reasonable to assume that educational experiences, both at the parental and child levels, might have impacted the outcomes observed in our study and should be considered as covariate in further research.

More than 80% of the refugee group consisted of families from Afghanistan, which may limit generalizability to other refugee populations. As a clinical sample, the comparison group represents a high-risk population that is not reflective of the general population, thereby emphasizing the relevance of our presented findings, especially regarding group differences in children’s social play. However, literature suggests that children with developmental impairments show play development comparable to healthy, normally developing children [32]. Moreover, the comparison group also consisted of many families with migration background as a shared characteristic with the refugee group. Therefore, we can still draw meaningful conclusions from our findings and contribute to the understanding of play development in young children.

### Implications for Research and Practice

Our study highlights the importance of standardized play observations when assessing young children’s mental health and development, particularly in low-resource refugee settings where other commonly used tests may not be valid or meaningful. As a non-invasive, culture-fair, and strength-based measure it allows clinicians and researchers to make a more accurate assessment of children’s strengths and resources, counteracting stereotypical perceptions and discrimination of refugee populations [20] and allowing for more targeted support and positive therapeutic treatments [15]. To further enhance the validity of our standardized play observations, future studies should compare the effectiveness of play measures incorporating shorter and longer play sequences and considering additional indicators like play interruptions, children’s emotional state, and aggressive sequences during play [10, 12]. Given that forced displacement in and of itself is not only an adverse experience, but also increases the likelihood of encountering further adversities in different stages of migration, future studies should address the complex effects of displacement related experiences on various aspects of child development and play.

## Conclusion

Our play observations showed that high level of adverse experiences and parental distress have a detrimental effect on the mental health and development of young children, especially for refugee children who are more likely to face restricted access to play-enhancing environments and stable settlement. We advocate for policies and practices that prioritize children’s need for safe play spaces and foster supportive parent work for refugee families soon after arriving in the host country.

### Summary

Our study aimed to evaluate the effectiveness of systematic play observations in assessing the mental health and development of young children. Our results provided valuable insights, indicating that children with more parent-reported adverse experiences showed less social-interactive play. Further, group comparisons yielded differential results for children with and without refugee experience: in the clinical comparison group born and raised in Germany, play variables were significantly correlated with IQ-testing scores, learning performance, and vocabulary, whereas social play correlated with educator-rated social-emotional competencies, parental distress and time spent in Germany in the refugee group. These findings do not only highlight the detrimental effects of adverse experiences and parental distress on young children’s play behavior, mental health, and development, but also show that our standardized play observation offers a child-centered and culturally sensitive diagnostic tool, particularly useful for refugee children where parent- or educator ratings are often not available and common assessment methods as IQ-, learning and language tests might not be applicable or valid due to language and cultural gaps.

## Data Availability

Deidentified individual participant data will be made available, in addition to study protocols, the statistical analysis plan, and the informed consent form. The data will be made available upon publication to researchers who provide a methodologically sound proposal for use in achieving the goals of the approved proposal.

## Abbreviations

PTSD: Post-traumatic stress disorder
SDQ: Strength and Difficulties Questionnaire [26]
VBV: Behavior Observation Scale for 3- to 6-year-old children [17]
KABC-II: Kaufmann Assessment Battery for Children [36]
IQ: Intelligence Quotient
